# Seroprevalence and immunity of SARS-CoV-2 infection in children and adolescents in schools in Switzerland: design for a longitudinal, school-based prospective cohort study

**DOI:** 10.1007/s00038-020-01495-z

**Published:** 2020-10-15

**Authors:** Agne Ulyte, Thomas Radtke, Irène A. Abela, Sarah R. Haile, Julia Braun, Ruedi Jung, Christoph Berger, Alexandra Trkola, Jan Fehr, Milo A. Puhan, Susi Kriemler

**Affiliations:** 1 Epidemiology, Biostatistics and Prevention Institute (EBPI), University of Zurich, Hirschengraben 84, 8001 Zurich, Switzerland; 2 Institute of Medical Virology, University of Zurich, Zurich, Switzerland; 3 Division of Infectious Diseases, University Children’s Hospital Zurich, Zurich, Switzerland

**Keywords:** SARS-CoV-2, COVID-19, Children, Adolescents, School, Protocol

## Abstract

**Objectives:**

This longitudinal cohort study aims to assess the extent and patterns of seroprevalence of severe acute respiratory syndrome coronavirus 2 (SARS-CoV-2) antibodies in school-attending children, and their parents and school personnel. It will examine risk factors for infection, the relationship between seropositivity and symptoms, and temporal persistence of antibodies.

**Methods:**

The study (*Ciao Corona*) will enroll a regionally representative, random sample of schools in the canton of Zurich, where 18% of the Swiss population live. Children aged 5–16 years, attending primary and secondary schools, and their parents and school personnel are invited. Venous blood and saliva samples are collected for serological testing in June/July 2020, in October/November 2020, and in March/April 2021. Bi-monthly questionnaires will cover SARS-CoV-2 symptoms and tests, health, preventive behavior, and lifestyle information. Hierarchical Bayesian logistic regression models will account for sensitivity and specificity of the serological tests in the analyses and complex sampling structure, i.e., clustering within classes and schools.

**Results and conclusions:**

This unique school-based study will allow describing temporal trends of immunity, evaluate effects of preventive measures and will inform goal-oriented policy decisions during subsequent outbreaks.

***Trial registration*** ClinicalTrials.gov Identifier: NCT04448717, registered June 26, 2020. https://clinicaltrials.gov/ct2/show/NCT04448717.

**Electronic supplementary material:**

The online version of this article (10.1007/s00038-020-01495-z) contains supplementary material, which is available to authorized users.

## Introduction

Decisions on school openings or closures during the severe acute respiratory syndrome coronavirus 2 (SARS-CoV-2) pandemic vary greatly across and within countries. While some countries kept schools mostly open (e.g., Sweden, Australia) or reopened early (e.g., Denmark), others opted for prolonged closures with decisions on reopening pending (e.g., the USA, Italy, Ireland). Early school closures in response to the pandemic were partly guided by evidence of transmission of other viruses, such as influenza (Cauchemez et al. 2009; Litvinova et al. 2019), but the current reports suggest that the susceptibility and transmissibility of children may be largely different for SARS-CoV-2 (Viner et al. 2020). Lower prevalence of SARS-CoV-2 infection is reported in younger children, which could potentially be explained by less frequent infection or underestimation due to more frequently asymptomatic infection course (Stringhini et al. 2020; Gudbjartsson et al. 2020; Pollán et al. 2020). Although children may infect others less often than adults, their exact role in transmission pathways is still not clear (Lee and Raszka 2020).

Several studies have reported population-level seroprevalence of SARS-CoV-2, including the subpopulation of children, reflecting the cumulative incidence proportion of the infection (Stringhini et al. 2020; Pollán et al. 2020). Such studies focused on households, leaving the roles of schools in SARS-CoV-2 transmission unclear, especially as they were often conducted during school closures. As most of school-aged children and adolescents’ social interactions take place in family and school (Mossong et al. 2008), schools could play a crucial role in spreading the infection. However, most evidence of SARS-CoV-2 infection spread in schools currently comes from anecdotal reports and case studies (National Centre for Immunisation Research and Surveillance (NCIRS) 2020; Fontanet et al. 2020).

Many schools were or are still closed worldwide in response to the pandemic, without solid arguments for or sufficient understanding of potential consequences of school closure policies (Masonbrink and Hurley 2020; Silverman et al. 2020). 1.6 billion learners worldwide (90% of all) were affected by school closure (UNESCO 2020). In the USA, school closures were coordinated on state and district level, with all USA public schools eventually closing from March 25, 2020 (Education Week 2020). In Switzerland, schools were closed from March 16 to May 10, 2020 (switching from regular in-person interaction to home and online schooling), then partly reopened until June 7 (e.g., teaching in half-classes, restricting larger group activities), when regular teaching resumed again (Swiss National COVID-19 Science Task Force 2020).

There is an urgent need for representative, population-based studies on children and adolescents, especially in school settings, to answer questions about prevalence, infection routes, asymptomatic cases, risk factors, and duration of immunity to SARS-CoV-2 infection. This article reports on the design and protocol of a longitudinal school-based seroprevalence study conducted in Zurich, the largest canton of Switzerland. The study is part of a larger nationally coordinated research network *Corona Immunitas*, and one of the first and largest representative studies of SARS-CoV-2 spread in children and adolescents in schools, globally.

## Methods

### Study overview, design and population

#### Study objectives

The study focuses on the seroprevalence and potential clustering of SARS-CoV-2 infection in children and adolescents attending school, as well as history, symptoms, and risk factors for SARS-CoV-2, health, lifestyle, and quality of life outcomes. It aims to address the following objectives:

1. To repeatedly determine the seroprevalence of SARS-CoV-2 antibodies in school-aged children covering grades one to eight (approximately 6–16 years old) in June/July 2020 (after the semi-lockdown and the subsequent reopening of schools), in October/November 2020 (3 months after the start of the next school year), and in March/April 2021 (after winter).
2. To examine clustering of seropositive cases within classes, schools and districts, and temporal evolution of the clusters.
3. To determine the proportion of asymptomatic children and adolescents with SARS-CoV-2 antibodies.
4. To determine the duration of the acquired immunity by examining new infections in children with positive serology and temporal persistence of detectable SARS-CoV-2 antibodies.
5. To identify sociodemographic, exposure, hygiene, school- and family-based behavioral and environmental risk factors for SARS-CoV-2 infection;
6. To assess how school-children and their families adjust their lives and adopt preventive measures for SARS-CoV-2 over extended periods of time.
7. To assess how quality of life is affected by the epidemic and preventive measures imposed or recommended by health authorities.
8. To assess how schools adopt preventive measures for SARS-CoV-2 infection over extended periods of time, and how they influence the infection rate;
9. To assess seroprevalence, clustering, and possible routes of transmission to and from children, school personnel, and parents.

#### Study design

This is a longitudinal, population-based observational study in a regionally representative cohort of children and adolescents from randomly selected schools and classes in the canton of Zurich, Switzerland. The study is embedded in a Swiss-wide research program *Corona Immunitas* (www.corona-immunitas.ch), where another 25’000 persons (mostly adults) will be enrolled in over 20 prospective studies with fully aligned study protocols (West et al. 2020).

Children participants were enrolled from June 16 to July 9, 2020, whereas parents and school personnel are enrolled from August 20 to September 5. The follow-up of the enrolled classes of children, their parents, and school personnel is planned until April 2021.

The longitudinal design allows for monitoring the evolution of the epidemic, as well as the impact of school-based and other preventive measures. We pre-defined three phases, with the possibility for adaptation (i.e., adding Phase IV) according to the dynamic of the pandemic:

- Phase I (June to September 2020): Baseline estimate of seroprevalence of SARS-Cov-2 in school-children, parents, and school personnel shortly after the lockdown and subsequent reopening of schools.
- Phase II (October/November 2020): Estimate of seroprevalence in the same cohort of children after the summer holiday and 3 months of school.
- Phase III (March/April 2021): Estimate of seroprevalence in the same cohort of children, parents and school personnel after the winter season.

#### Study setting: primary and secondary schools in Switzerland

**Fig. 1:**
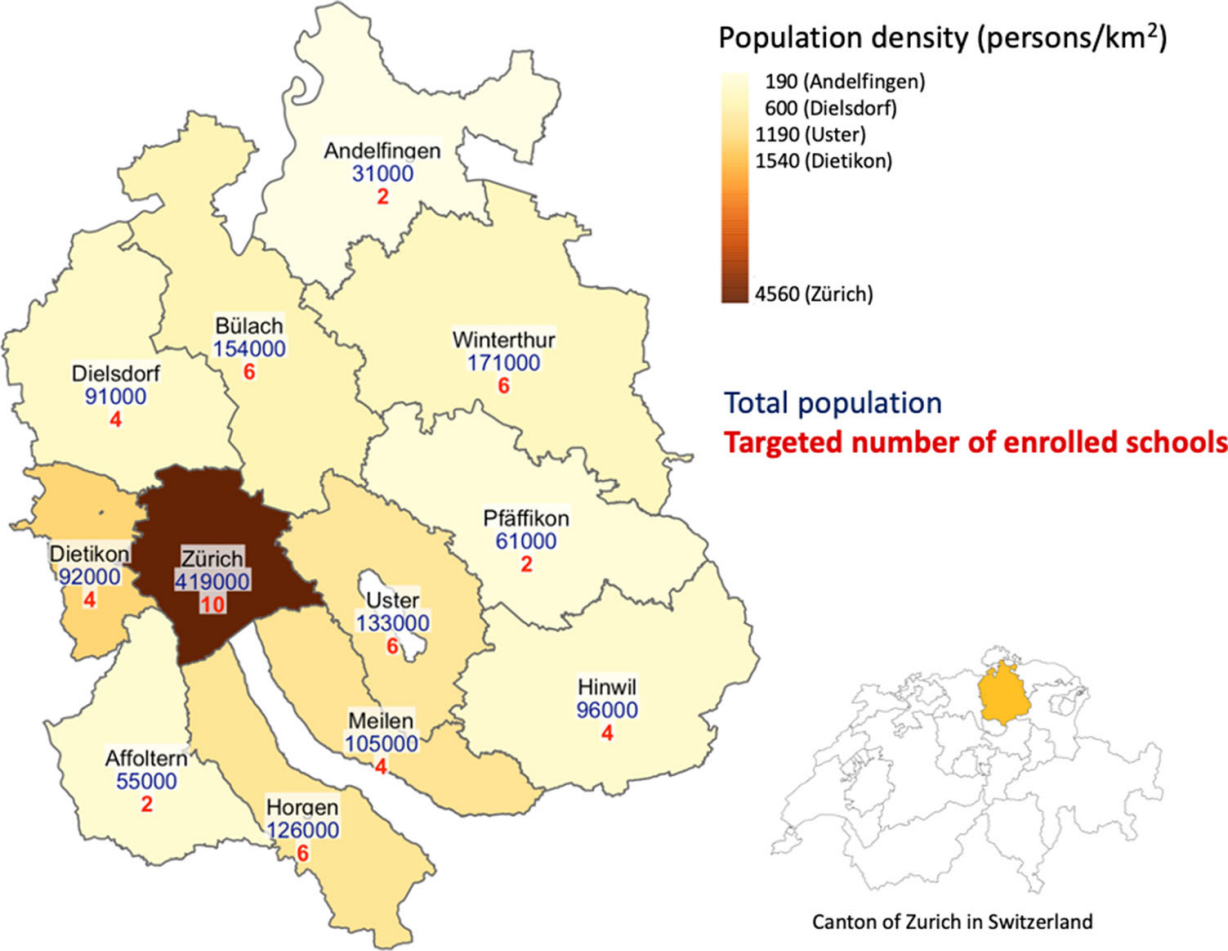
Districts of the canton of Zurich: population density, count, and targeted number of enrolled schools (Switzerland, 2020)

One out of six (1.5 million) inhabitants of Switzerland live in the canton of Zurich. The canton is divided into 12 districts (Fig. 1). Primary school is divided into lower level (“Unterstufe”) with grades 1–3 (kindergarten not included in this study) and middle level (“Mittelstufe”) with grades 4–6. Secondary school comprises upper level (“Oberstufe”) with grades 7–9. A significant proportion of schools, particularly in rural setting, adopt *age*-*mixed learning* methodology, in which students of two or three adjacent grades are taught in the same classroom.

Further details on population characteristics and the school system are provided in the online data supplement.

#### Ethical aspects

The study was approved by the Cantonal Ethics Committee Zurich (2020-01336). Written informed consent is obtained from parents or legal guardians (referred to as parents further on) of participating children. Children aged 14 years and older can confirm the consent themselves. Additional informed consent is obtained for biobanking plasma samples for subsequent testing within the scope of SARS-CoV-2 seroprevalence studies.

#### Schools: sampling and sample size

We stratified the random selection of schools within districts of the canton, and random selection of classes was stratified within lower, middle, and upper levels of schools. All children attending the selected classes are invited, except in mixed-age classes (in which only students from the eligible grades are invited).

We selected the primary schools randomly and matched the closest secondary school geographically. The targeted number of schools to enroll per district ranges from 2 to 10, depending on the district size. After the initial invitation round, overall school participation rate is assessed and additional schools are selected within required districts, until the aimed number is reached, or further recruitment would not be feasible. Population sizes and targeted number of enrolled schools within districts are depicted in Fig. 1.

The overall targeted number of schools is 58 (29 primary and 29 secondary schools). We aim to invite at least 3 classes and at least 40 children per school level (lower and middle in primary, upper in secondary schools). We will reassess the number of classes and children to invite after calculating the average children participation rate in the first week of enrollment. If needed, additional classes will be invited, aiming to enroll at least 40 children per school level. Assuming a participation rate of 60–80% per class, we would enroll 2100–2800 children in June/July 2020. We expect a seroprevalence rate of 1–5% based on the existing research (Stringhini et al. 2020). Depending on the specificity and sensitivity parameters of the test, we expect a precision of about ± 2%.

#### Population: definition

Children and adolescents residing in Switzerland and attending a selected public or private, primary or secondary school (approximate age 6–16 years) in the canton of Zurich are eligible for the study, in addition to their parents living in the same household, and the entire personnel of participating schools. Main exclusion criteria for schools are small school size (< 40 children in a selected school level), and for participants—suspected or confirmed infection with SARS-CoV-2 during testing. School grades 1–2, 4–5, and 7–8 are included; grades 3, 6, and 9 grades are excluded as these students may move to another school after the summer break, and follow-up would be compromised. In age-mixed learning classes in primary schools, only grades 1 and 5 are included as children in other grades potentially change the class after the summer break. Detailed inclusion and exclusion criteria are provided in the online data supplement.

### Study procedures

#### Recruitment and study timeline

**Fig. 2:**
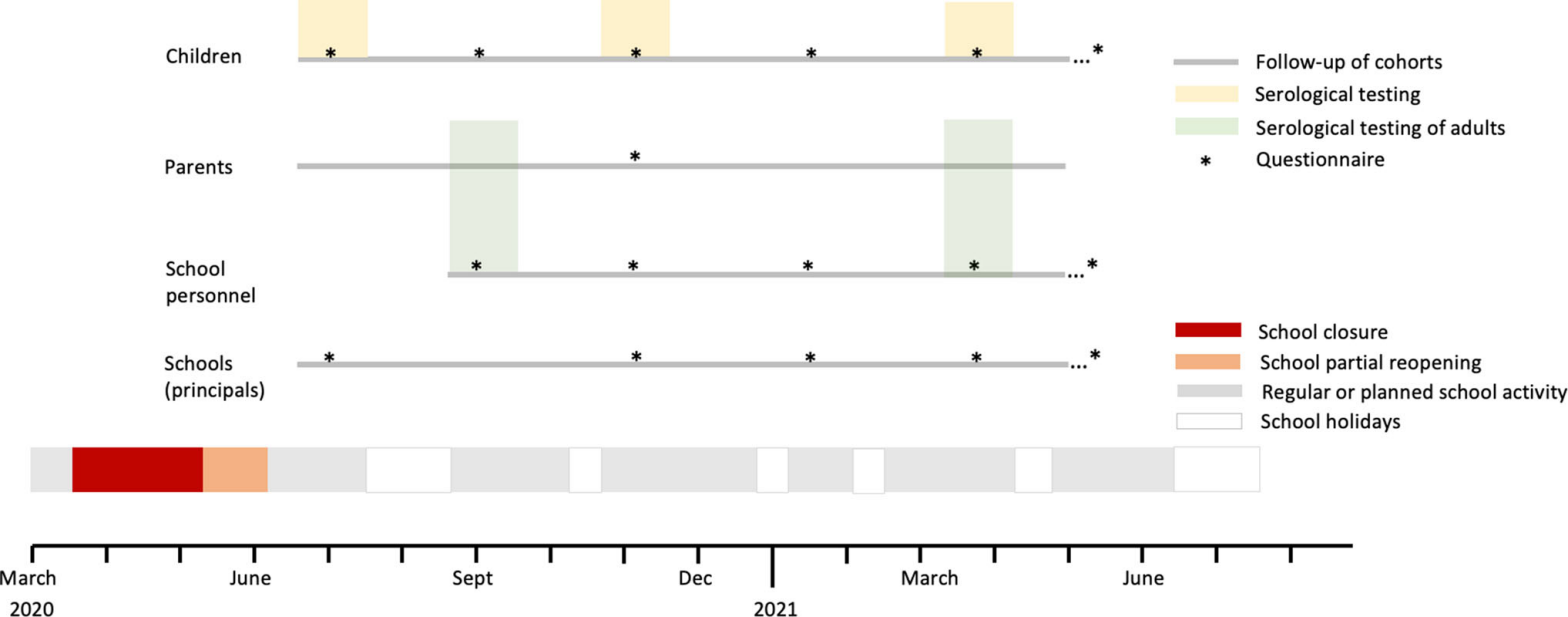
Timeline of study recruitment, serological testing, and follow-up (*Ciao Corona* study, Switzerland, 2020–2021). Note that the children’s questionnaire includes questions for parents. Timing of testing and questionnaires is approximate and will depend on the development of the pandemic

The process of recruitment and testing of children, their parents and school personnel is depicted in Fig. 2. Randomly selected schools receive an email from the study group, including study information, a link to the study website (www.ciao-corona.ch), informational videos in multiple languages for schools, parents, and children. Further details on the recruitment process are provided in the online data supplement.

#### Collection of samples and testing at schools

At each of the planned testing phases, the study team will come to the participating school for a half or full day, depending on school size. Testing will take place in a sufficiently large room, in small groups of participating children, with all necessary hygiene and distancing precautions. First, information is provided, child’s identity and consent confirmed, and saliva collected. Venous blood samples will be collected at supine position with the help of anesthetic patches, applied 45–60 min prior to venipuncture. Participating children receive a small age-appropriate gift (worth $5–20) at each testing.

We will invite adult participants for testing in schools or at a testing center. Venous blood will be collected. Collected samples are stored, cooled and transported to the laboratory daily after the testing is finished.

#### Measurements

Summary information of specimens and questionnaires collected is provided in Table 1.

**Table 1:** Summary of testing and measurements in study populations (*Ciao Corona* study, Switzerland, 2020–2021)

Measurement	School principals	Children			Parents		School personnel	
	Questionnaire	Serological testing	Baseline questionnaire	Follow-up questionnaire	Serological testing	Questionnaire	Serological testing	Questionnaire
Frequency	5 times	3 times	3 times^a^	Bi-monthly	2 times	Once	2 times	Bi-monthly
Specimen								
Venous blood		X			X		X	
Saliva		X^b^						
Collected information								
School structure	X							
Preventive measures	X		X	X		X		X
Sociodemographic			X			X		X
SARS-CoV-2 symptoms and diagnosis in households	X		X	X		X		X
Household composition			X					X
Quality of life			X					X
Lifestyle			X					

^a^Full questionnaire at baseline, shortened version at follow-up

^b^Depending on the results of the validation study; SARS-CoV-2, severe acute respiratory syndrome coronavirus 2

#### Blood and saliva samples for serological testing

For each child and adult participant, one sample (7-9 mL for children and 4.9 mL for adults) of venous blood will be collected for the assessment of SARS-CoV-2 antibodies. Plasma will be separated, aliquoted into 1 mL tubes and biobanked at -20 °C until testing.

Saliva samples are collected in clean tubes and enriched with virus transport medium. The validity of serological SARS-CoV-2 testing in saliva is currently not confirmed; therefore, saliva will first be validated for serological testing. If testing in saliva is deemed sufficiently accurate in comparison with testing of blood, venous blood sampling might not be necessary in further testing phases. Conversely, if serological testing in saliva does not offer sufficient accuracy, saliva sample collection will not be continued.

For serological analysis, one or both of the following tests will be used depending on availability, utilization in the other nationally coordinated research program *Corona Immunitas* studies and thus ability to compare the results, and the cross-validation of the results. The first option is an in-house developed bead-based binding assay based on the Luminex technology. The ABCORA test (version 2.0) provides a highly differentiated picture of the immune response: immunoglobulins G (IgG), M (IgM), and A (IgA) antibodies against four SARS-CoV-2 targets (receptor binding domain (RBD), spike proteins S1 and S2, and the nucleocapsid protein (N) of SARS-CoV-2) are analyzed, resulting in twelve analyzed parameters. Owing to the broad assessment of serological parameters, the ABCORA 2.0 test provides an estimate of infection recency. Based on the ABCORA 2.0 results, the seroconversion status of a sample will be classified as positive, weakly reactive, indeterminate, or negative, based on pre-specified threshold values of detected antibody reactivities. In a validation study (unpublished) of 104 samples of SARS-CoV-2 reverse transcription polymerase chain reaction (RT-PCR) positive persons and 251 samples of pre-pandemic, healthy blood donors, the test had sensitivity of 93.3–95.2% (compared to sensitivity of 88.5–93.3% in commercially available tests) and specificity of 98.4–99.6% (depending on the threshold definition of positive and negative cases).

The second option is the SenASTrIS (Sensitive Anti-SARS-CoV-2 Spike Trimer Immunoglobulin Serological) assay developed by the Centre Hospitalier Universitaire Vaudois (CHUV), the Swiss Federal Institute of Technology in Lausanne (EPFL) and the Swiss Vaccine Center (Fenwick et al. 2020). The test has demonstrated 94.0% sensitivity and 99.2% specificity for detection of SARS-CoV-2 IgG antibodies, and up to 98.3% sensitivity and 98.4% specificity with a combined IgG and IgA test. The test is used by all study sites of *Corona Immunitas*.

#### Collection of questionnaire data

Study participants and parents of the participating children together with the child will fill baseline and follow-up questionnaires online (when necessary, on paper or over phone) (see Fig. 2). We will send follow-up questionnaires approximately bi-monthly, adapting to the school year timing, at least until April 2021.

The following baseline information is collected for all participants (Table 1): sociodemographic and basic health information; number of people in the household; SARS-CoV-2 infection and testing related information for participant and their household; exposures and preventive behavior related to the pandemic within the household since January 2020; quality of life parameters. In addition, for the children, lifestyle, mental health and well-being information is collected. A shortened questionnaire will be repeated at the subsequent testing phases.

Bi-monthly follow-up of participants (Table 1) will assess flu-like symptoms (onset, type, duration) and use of related health-care services within the household; SARS-CoV-2 infection test results outside this research study within the household; adherence to preventive measures and possible exposures (travels abroad, contact with confirmed SARS-Cov-2 cases, etc.) within the household.

School principals will fill in questionnaires at and between testing phases. The following information will be collected: total number of children per school and school level; number of children and teachers in classes; preventive measures at school organizational, infrastructure and personnel levels. Socioeconomic status of the school will also be estimated at baseline from official statistics.

More information on data collected with each questionnaire is provided in the online data supplement.

### Study data

#### Data management

We will collect study data in REDCap (Research Electronic Data Capture), a secure, web-based application with access restricted to selected study personnel. The database will also be used to send out online surveys to school personnel and parents and deliver study results per email. Further details are provided in the online data supplement.

#### Data analysis

We will perform descriptive analysis of participant sociodemographic, lifestyle, and behavior information. Total seroprevalence and cumulative incidence (i.e., total number of RT-PCR-confirmed infections in official statistics per population) will be calculated and compared, as well as age-, time-, and region-specific estimates. In order to also include the sensitivity and specificity of the serological test in the analyses and account for the complex sampling structure (clustering within classes and schools), we will use hierarchical Bayesian logistic regression models (Stringhini et al. 2020). The total numbers of school children in the respective grades per district will be used for post-stratification, so that the estimates are representative for the demographics of the canton of Zurich.

We will assess the associations with health and quality of life outcomes with multiple regression models. Other planned estimates include proportion of seropositive individuals who have been asymptomatic, risk factors for infection at individual and school level. We will also assess associations of levels of IgG, IgM, and IgA antibodies with symptoms and risk factors.

#### Patient and public involvement

Several school principals were consulted during the development of the protocol to ensure feasibility of the planned study procedures. Early feedback was collected from children and parents invited to participate, in order to adapt the communication strategies and channels. Further feedback was collected from enrolled children and school principals during the first testing phase, in order to adapt subsequent testing phases and adult testing. Numerous online informational sessions, encouraging open exchange and feedback, were organized for school principals, personnel, and parents of the children. Results of individual tests will be communicated to the participants, and overall study results disseminated to participating schools. Findings will be disseminated in lay language in the national and local press, to the national and regional educational and public health departments, and via the website of the study.

## Discussion

This longitudinal population-based cohort study is unique for its focus on children and adolescents in schools. Major policy decisions on temporary school closures or schedules have been implemented globally in response to the pandemic, despite the lack of knowledge how schools contribute to the spread of SARS-CoV-2 infection. This study will contribute to the evidence required to define the necessary and sufficient preventive measures to balance infection control and impact of school closure.

Currently available population-based seroprevalence studies, which have included children, were mostly conducted in household settings. In May 2020, only 0.8% of children aged 5–9 years were seropositive for SARS-CoV-2 in Geneva, Switzerland, in contrast to 9.6% of children and adolescents aged 10–19 years and 9.9% of adults aged 20–49 years (Stringhini et al. 2020). In April and May 2020, 3.8% of children and adolescents aged 0–19 years were seropositive in Spain, compared to 4.5–5.0% in older age groups (Pollán et al. 2020). In contrast, the current study will primarily consider schools. By analyzing seroprevalence on individual, class, and school levels, as well as in parents and school personnel, we will be able to identify clusters within these structures. Such knowledge could help to decide if individual classes or whole schools need to be closed to prevent SARS-CoV-2 infection spread. In addition, testing the entire school personnel will contribute evidence on which employees at schools are the most susceptible to infection.

Only few related planned or ongoing studies have been reported worldwide. In the UK, a study run by Public Health England aims to test seroprevalence in child care facilities and schools in England in May/June, July, and end of autumn 2020 (Ladhani et al. 2020). However, it is not clear if the sampling of schools will be random or stratified by region or if the structure within schools (classes) will be considered. A smaller study in Berlin, Germany, aims to test 24 randomly selected schools, including 20–40 children and adolescents (500–1000 in total) and 5–10 staff members at each school (Charité 2020).

The results of this study will likely be generalizable to other cantons in Switzerland and internationally, particularly to high- and middle-income countries. The canton of Zurich includes both urban and rural settings, as well as an ethnically and linguistically diverse population. Although the rates of seroprevalence are always location- and time- specific, we believe that the longitudinal design will allow us to investigate many stages of the pandemic. The wide age range of the participants (6–16-year-old) will also allow to detect age-based variation in seroprevalence. This variation can be expected because risk factors, natural history of the infection, and transmission routes might vary by age.

### Limitations

The study faces a few challenges and limitations. First, high participation rate of schools and children is required for sufficient power to analyze different regions and clusters within classes and schools. We believe that the high public interest will lead to increased participation, which could otherwise be rather limited in a study collecting venous blood samples in children. In fact, in the initial testing phase in June/July 2020, 55 schools and more than 2500 children were successfully enrolled. Second, the protocol of the longitudinal study will have to be flexible as the pandemic and serological testing methods develop. For this reason, the specific time points of serological testing cannot be fixed in advance (e.g., in case of school closure during the course of the study). Similarly, in further testing phases, serological testing in saliva or blood might be sufficient, depending on the outcome of the validation of serological testing in saliva. In addition, in order to recruit a sufficient number of schools still before school summer holidays, three rounds of invitations were needed, leading to potential over- or under-sampling of schools in certain districts. However, the sampling discrepancies can be adjusted with weighing of results. Finally, although the accuracy of the serological tests that will be used in this study is even superior to the best commercially available alternatives, the general knowledge on the validity and clinical interpretation of the tests is still incomplete—which is, partly, the motivation for this study. The interpretation of the serology results will develop as evidence is accumulated on the clinical and immunological features of SARS-CoV-2 antibodies.

### Conclusions

This population-based cohort study with randomly selected schools and classes across the age range for mandatory school time offers a unique opportunity to observe the longitudinal spread of SARS-CoV-2 infection in children in schools, as well as in their parents and school personnel, thus studying the whole school community. We will report SARS-CoV-2 seroprevalence in children by age groups and regions, provide essential information on possible transmission routes and immunity over time, and assess individual and school-level risk factors for infection. The longitudinal design will allow describing temporal trends of immunity to SARS-CoV-2 and evaluating effects of school structure and preventive measures. The results of the study will inform goal-oriented policy decisions in school management during subsequent outbreaks.

## Electronic supplementary material

Below is the link to the electronic supplementary material.

Supplementary material 1 (DOCX 34 kb)
