# Supplementary material for: Seroprevalence and immunity of SARS-CoV-2 infection in children and adolescents in schools in Switzerland: design for a longitudinal, school-based prospective cohort study
Source: Int J Public Health. 2020 Oct 15;65(9):1549–57. doi: 10.1007/s00038-020-01495-z (PMC7561232; doi:10.1007/s00038-020-01495-z)
Supplement: Supplementary file 1 — Supplementary material 1 (DOCX 34 kb) [file 38_2020_1495_MOESM1_ESM.docx]

**International Journal of Public Health**

**DATA SUPPLEMENT**

**Seroprevalence and immunity of SARS-CoV-2 infection in children and adolescents in schools in Switzerland: design for a longitudinal, school-based prospective cohort study**

Agne Ulyte*, Thomas Radtke*, Irène Abela, Sarah Haile, Julia Braun, Ruedi Jung, Christoph Berger, Alexandra Trkola, Jan Fehr, Milo A. Puhan, Susi Kriemler

* shared first authorship

# **Methods**

**Study setting: primary and secondary schools in Switzerland**

One out of six (1.5 million) inhabitants of Switzerland live in the canton of Zurich. The canton is divided into 12 districts (see Figure 1 in the original manuscript). The population size and density within districts is very heterogeneous and ranges from 32’000 inhabitants (188 inhabitants per km^2^) in the district Andelfingen to 423’000 inhabitants (4’732 per km^2^) in the city of Zurich. The canton of Zurich is also culturally and linguistically highly diverse. Approximately 25% of cantonal population is non-Swiss, with even higher proportion in the city of Zurich (32%). Approximately 80% of people speak German or Swiss-German as their main language, 8% English, 6% Italian, followed by Albanian, French and Serbo-croatian^1^.

In the canton of Zurich, 140’000 children attend primary or secondary schools, covering ages from 5 to 16 years, with 93% of children enrolled in public schools^2^. Primary school is divided into two levels – lower (“Unterstufe”) with grades 1-3 (kindergarten not included), and middle (“Mittelstufe”) with grades 4-6. Secondary school comprises upper level (“Oberstufe”) with grades seven to nine. A significant proportion of schools, particularly in rural setting, adopt *age-mixed learning* methodology, in which students for two or three adjacent grades are taught in the same classroom.

**Study population: inclusion and exclusion criteria**

Children and adolescents residing in Switzerland and attending a randomly selected public or private, primary or secondary school (approximate age 5 to 16 years) in the canton of Zurich are eligible for the study. On the class level, 1^st^-2^nd^, 4^th^-5^th^ and 7^th^-8^th^ grades are included. Third, sixth and ninth grades are excluded as they potentially move to another school in the next school year, and follow-up would be compromised. In age-mixed learning classes in primary schools, only first and fifth grades are included as children in other grades potentially change the class in the next school year.

Exclusion criteria are: for children – attendance of kindergarten grades (due to difficulties in enrolling, collecting venous blood samples, and following up the cohort); for all participants – any acute respiratory infection with symptoms present within the last 48 hours before the testing at school; and severe acute respiratory syndrome coronavirus 2 (SARS-CoV-2) infection confirmed with reverse transcription polymerase chain reaction (RT-PCR) and symptom onset within the last 21 days from testing or persistence until within the last 48 hours before the testing.

On the school level, public and private, primary or secondary schools in the canton of Zurich, randomly selected and consenting to participate are included. Excluded are schools with <40 students in one of the sampled levels, as the expected number of enrolled children would be too low for feasibility, and school for children with special needs.

**Study procedures**

**Recruitment and study timeline**

The process of recruitment, communication with invited schools, children and their parents, and testing, is depicted in Figure 2 in the original manuscript.

Randomly selected schools receive an email from the study group, including study information, link to study website (www.ciao-corona.ch), informational videos in multiple languages (for schools and parents: <https://youtu.be/9ITy7oS6FhQ>, for children: <https://youtu.be/C9sKOop93so> and <https://youtu.be/lhIX377uvzk>), and invitation to an online meeting, is sent to the school authorities. Further details are provided in the online data supplement.

After accepting to participate, schools receive further information via email and on paper, to be distributed to children and parents of the selected classes. School authorities and parents are invited to online meetings before enrollment, and can contact study team via two dedicated phone lines and an email address.

Consenting schools are scheduled for baseline testing before summer holiday (June 16 – July 9, 2020) and subsequently for testing in autumn 2020 and winter 2021. Written consents for children are collected by class teachers before the testing. Parents receive a personal access code to an online questionnaire. In case the questionnaire is not filled in a few days after the testing at school, they are reminded by email or phone. Personal translation support into eight commonly spoken languages (French, English, Italian, Spanish, Portuguese, Albanian, Turkish, Tamil) is offered for the questionnaires. Parents of participating children are invited to the study with personal emails and school personnel with an email of school principals including a link to registration survey. Invited adults are sent detailed study information at least a few days in advance of the testing.

Parents and school personnel will be tested after school summer holidays (August/September 2020). Parents will be tested only once, as the study focuses rather on the community within schools. School personnel will further be tested at two additional time points together with the cohort of children. Children in the selected classes, who did not take part in the first phase of testing (June/July 2020), will be given an opportunity to enroll during the second phase. All withdrawals and their reasons are documented.

**Participant recruitment and communication**

Once a school is selected, an email is sent to the school authorities, including study information, link to study website, informational videos, and invitation to an online meeting. Schools are requested to respond within 3-5 working days about their willingness to participate or reasons for declining. Participating schools receive a study package on paper (checklist for schools, information, consent and questionnaire filling instructions for children and parents), to be distributed to the teachers and children of the selected classes.

Consenting schools are scheduled for baseline testing before the summer holiday (June 16 – July 10, 2020). Subsequent testing in autumn 2020 and Winter 2021 will be scheduled a few weeks before the testing period. Written consents of the children and their parents are collected by class teachers in closed envelopes before the baseline testing date. Contact information of consenting parents is provided by schools, in case of emergency during the testing.

Parents receive a personal access code to online baseline questionnaire. In case the questionnaire is not filled in a few days after the testing at school, email reminders are sent. In case the questionnaire is not filled in by the end of testing period, parents are contacted by phone, offering to fill in the questionnaire on the phone or on paper, or personal translation support into 8 commonly spoken languages in the canton of Zurich (French, English, Italian, Spanish, Portuguese, Albanian, Turkish, Tamil).

Children in the selected classes, who did not take part in the first phase of testing (June/July 2020), will be given an opportunity to be enrolled during the second phase. Study information will be sent to these children through school principals again before the second phase of testing.

Parents of participating children will be invited via personal emails (collected in the baseline questionnaires for children and parents). School personnel will be invited through an email of school principals, including study information. Invited adults will be sent detailed study information at least 24 hours in advance of the testing, and will have the opportunity to ask questions via dedicated phone lines and email address.

All withdrawals are documented and the reason for withdrawal recorded in the database. Withdrawals will be discussed in the final publication of the study. No attempt is made to replace study participants who withdraw from the study.

Information about the study, testing and participation is communicated via multiple channels both to schools and to participating children and parents, including email, phone and in person. School authorities and parents are invited to attend online meetings with the study team before enrollment, and can contact study team via two dedicated phone lines and email address. Three versions of study information are prepared to accommodate parents with different levels of German proficiency (official 10-page, three-page and one-page summary information). Short videos about study design and testing procedure (for schools and parents: <https://youtu.be/9ITy7oS6FhQ>, for children: <https://youtu.be/C9sKOop93so> and <https://youtu.be/lhIX377uvzk>) are available on the study website ([www.ciao-corona.ch](http://www.ciao-corona.ch)) with subtitles in eight most commonly spoken languages in the canton of Zurich. The website also features an infographic explaining SARS-CoV-2 infection and antibodies production

In case the questionnaire is not filled in by the end of testing period, parents are contacted by phone, offering to fill in the questionnaire on the phone or on paper, or personal translation support into 8 commonly spoken languages in the canton of Zurich (French, English, Italian, Spanish, Portuguese, Albanian, Turkish, Tamil). If necessary, personal support to fill in the online questionnaire will be offered during testing.

Individual serological test results will only be communicated to the participant and their family, and directly to the adult participants. Results as well as their interpretation are communicated via email (in case of negative results) or phone call (in case of positive results). Seroprevalence aggregated on school level is communicated to school principals as well as participating children and parents in the school.

**Collection of questionnaire data**

Baseline questionnaire for children will be filled by parents and children after enrollment in June/July 2020. The following information is collected: sociodemographic and basic health information of child and parents; number of people in the household; SARS-CoV-2 infection and testing related information of the child, parents and family in the same household; exposures and preventive behavior related to the pandemic within family and household since January 2020; lifestyle, mental health and well-being of the child. A shortened questionnaire will be repeated at the subsequent testing phases.

Short bi-monthly questionnaires will be sent until at least April 2021. The following information will be assessed: flu-like symptoms (onset, type, duration) and use of related health-care services within household; SARS-CoV-2 infection test results outside this research study within household; adherence to preventive measures and possible exposures (travels abroad, contact with confirmed SARS-Cov-2 cases, etc.) within household.

School principals will fill in questionnaires at and between each testing phase. The following information will be collected: total number of children per school and school level; number of children and teachers in classes; preventive measures at school organizational, infrastructure and personnel levels. Socioeconomic status of the school will also be estimated at baseline from official statistics.

Baseline questionnaire for school personnel participating in the study will cover sociodemographic, health and SARS-CoV-2 infection related information. SARS-CoV-2 infection and preventive measures related information will be collected bi-monthly.

## **Study data**

**Data management**

All study data will be collected in REDCap (Research Electronic Data Capture), a secure, web-based application with access restricted to selected study personnel. The database will also be used to send out online surveys to school personnel and parents, and to deliver study results per email. Further details are given in the data supplement. Data monitoring and plausibility checks will be done during the data collection period and prior to database closure. Following database closure, the exported database will be stored at a password-protected server at the University of Zurich.

Serology test results will be linked with questionnaire data for analysis, in a pseudonymized dataset with unique IDs, automatically created by REDCap. Data will be stored in de-identified format, with the linking key to personal identifiers stored securely with access restricted to the principal investigators of the study. Individual level data will be linked with the class, school, and region of school for hierarchical modeling.

**Supplemental references:**

S1. Statistics and Data. Canton of Zurich. . https://www.zh.ch/de/politik-staat/statistik-daten.html. Accessed July 14, 2020.

S2. Bista - Data Portal for Statistics of Education. Canton of Zurich. https://pub.bista.zh.ch/de/. Accessed July 16, 2020.
